# Wide-angle, wide-band, polarization-insensitive metamaterial absorber for thermal energy harvesting

**DOI:** 10.1038/s41598-020-73368-7

**Published:** 2020-10-01

**Authors:** Ahmed Elsharabasy, Mohamed Bakr, M. Jamal Deen

**Affiliations:** Electrical and Computer Engineering Department, McMaster University, Hamilton, ON Canada

**Keywords:** Energy science and technology, Energy harvesting, Renewable energy

## Abstract

We propose a wide-band metamaterial perfect absorber (MPA), using the coupling in the near-field of a quadruple split-ring resonator concentric with crossed ellipses. We designed the MPA with a metal–insulator-metal (MIM) structure for use in thermal energy harvesting. A gradient-based optimization approach was carried out to maximize the absorption of infrared (IR) radiation around 10 μm. Owing to the near-field coupling of resonators with optimal design parameters, the peaks of the absorption responses approach each other, thus broadening the overall bandwidth with almost unity absorptivity. The proposed design has a resonance at 10 μm resulting from magnetic polaritons (MPs) and thus maintains high absorption above 99% up to a range of incident-angles greater than 60° and exhibits a polarization-free behavior due to symmetry. When the optimal design was numerically examined to fabrication tolerances, it showed negligible sensitivities in the absorptivity with respect to design parameters. The strong electric field enhancement inside the split-ring gaps and between the ends of the cross arms and the surrounding ring enables designing MIM diodes to rectify the harvested thermal radiations at 288 K. MIM diodes can be built by the deposition of thin insulators to sit in these gaps. The MIM diode and MPA work together to harvest and rectify the incident IR radiation in a manner similar to the operation of rectennas. The MPA outperforms the traditional nano-antennas in impedance matching efficiency because of its higher resistance. Also, its dual-polarization reception capability doubles the rectenna efficiency. Our proposed MPA retained absorptivity more than 99% when coupled with MIM diodes whose resistances are in the range of 500 Ω–1 MΩ.

## Introduction

The current patterns of climate change have attracted considerable attention to the hidden costs of conventional energy sources such as fossil fuel. Renewable energy sources including solar, wind, geothermal and biomass have substantially less harmful impact on the environment by most measures^1^. One of the serious impacts of fossil fuels is global warming driven by increasing concentrations of greenhouse gas emissions^2^. These emissions decrease the thermal back-radiations leaving the atmosphere, thus leading to an energy imbalance between the earth and the sun and consequently warmer temperatures. The thermal energy emitted back from earth is considered an intact energy source in the infrared (IR) regime. The peak of these IR radiations corresponding to an average earth surface temperature of 288 K lies at wavelength of ~ 10 μm according to Wien's equation^3^.

Attempts to harvest the IR electromagnetic radiations with wavelengths around 10 μm, i.e. a frequency ~ 30 THz, were reported^4–9^. These efforts include using thermoelectric materials that can directly transform the thermal heat into dc voltages through the Seebeck effect^4,5^. However, their relatively high cost and low conversion efficiency at lower temperatures impedes their use at large scale^10^. A promising technique is the rectifying antennas (rectennas) which have been implemented successfully in the microwave regime with high efficiency^11^. The rectenna is comprised of a rectifying element which is the diode connected to the terminals of a receiving antenna designed to resonate around the peak of the incoming electromagnetic radiation^9^. The antenna can be considered as an absorbing element for the incoming electromagnetic radiation. It is capable of strongly enhancing the localized electromagnetic fields inside a tiny gap, i.e. hot spot, where the diode, the rectifying element, is situated. Metamaterials-based absorbers have been widely suggested for several applications including thermophotovoltaics^12^, spectroscopy^13^, and stealth technology^14^ due to their tunability for near unity absorption of electromagnetic radiations.

Metamaterials-based absorbers are engineered materials and structures that exhibit exotic properties in terms of absorption peaks and wavelengths through matching their equivalent permittivity and permeability with that of the surrounding medium^15–20^. A popular configuration of these metamaterials-based absorbers is the periodic metal–insulator-metal (MIM) structures^21,22^. The metal-dielectric interfaces allow the excitation of the plasmonic phenomenon which in turn manipulates the absorption at certain wavelengths to reach near unity where these MIM structures realize a nearly perfect absorber^22^. Therefore, building a metamaterial perfect absorber (MPA) with few nanometers gaps can replace the antenna in the rectenna device. The MIM-based MPA structure encompasses a repeatedly distributed metallic pattern at the top, another metallic layer in the bottom, and a thin dielectric film is sandwiched between these two metallic layers. The metallic patterns on top and the dielectric layer determine the number, peak and width of the absorption bands^23^, while the bottom metallic layer is to impede the transmission from occuring^24^.

The MIM-based MPA requires an ultra-fast diode that can follow the captured ultra-high frequency signal of the incident IR radiation at 30 THz^8^. MIM diodes offer this opportunity owing to their ultra-thin dielectric layers that result in tunneling as the dominant conduction mechanism^9^. The operation of the MPA in a rectenna for IR energy harvesting requires a small gap in the range of few nanometers to accommodate the MIM diode. One of the advantages of building MIM diodes within the MPA structure is the possibility of growing the dielectric layer in both structures simultaneously, which in turn simplifies the fabrication process.

In this work, we present metallic crossed ellipses (CE) concentric with a quadruple split-ring resonator (QSRR). The metallic top layer sandwiches a thin dielectric layer with the other metallic bottom layer. The MIM-based MPA structure is optimized to achieve near unity absorption with a wide-band response. This design can absorb IR radiation with both polarizations due to its symmetry and also offers a wide-angle of reception that surpasses the traditional antennas such as dipoles or bow-ties. Sensitivity analysis to the design geometry parameters shows insignificant change in the overall performance within the expected fabrication tolerances. The strong electric field enhancement inside all the gaps has broad bandwidth characteristics which in turn increase the amount of harvested IR radiation. The choice of Au/TiO_2_/Au and Au/TiO_2_/Ti allows for the use of simple fabrication techniques to build the proposed MIM structure. The proposed rectenna-like structure shows insensitive absorptivity to changes in the MIM diode’s resistance.

## MIM structure optimal design for IR energy harvesting

### Crossed-ellipses coincide with quadruple split-ring-resonator

**Figure 1:**
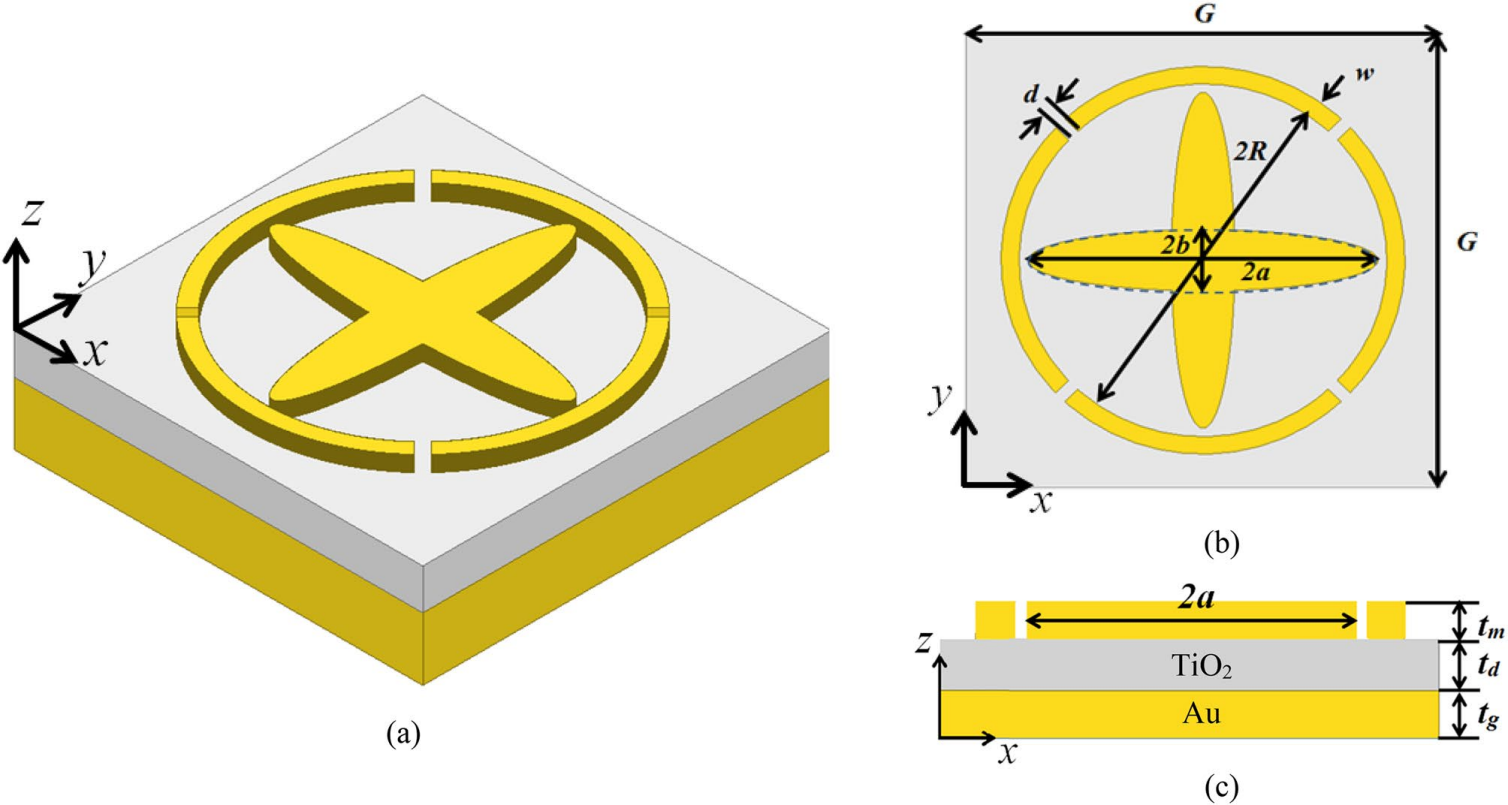
The proposed metamaterial perfect absorber (MPA) structure (**a**) 3D isometric view, (**b**) top view showing the design parameters of the split-ring-resonator (SRR) and crossed ellipses, (**c**) Cross-sectional view of the MPA showing the metal–insulator-metal (MIM) 3-layers with thicknesses as design parameters. (ANSYS HFSS 2018, https://www.ansys.com/).

First, the concept of building a perfect absorber that is compatible with MIM diodes integration through few-nanometers gaps was implemented. The selection of the quadruple split-ring-resonator (QSRR) successfully achieves near-unity absorption at 10 μm wavelength. In addition, the gaps of SRR formed 4 MIM diodes within the design. The arrangement of the designed perfect absorber combined with the diode resulted in a rectenna-like element capable of perfectly absorbing IR radiation and transforming it into useful DC power. To improve the rectification process, we designed crossed ellipses (CEs) concentric with the QSRR. The crossed ellipses exhibit an additional absorption band that is controllable through the major and minor axes of the ellipse. By adding this cross absorber, 4 more tiny gaps were formed within the inner perimeter of the SRR, which in turn double the rectification capability. A schematic diagram of one cell of the array of the metamaterial perfect absorber (MPA) based on a metal–insulator-metal architecture is shown in Fig. 1a. Gold is used to build the top resonators and the ground plane, while TiO_2_ is chosen to form the dielectric layer in between. The top view of the designed MPA showing the geometric parameters of both resonators is depicted in Fig. 1b. Additionally, the cross- section of the MPA is shown in Fig. 1c with the thicknesses for each layer as additional design parameters.

The initial dimensions of both QSRR and CEs were determined to form a wide-band absorber around 10 μm. The resonance wavelength of QSRR can be approximated analytically using an inductor-capacitor (LC) circuit model^25^:

1 $$ \mathop \lambda \nolimits_{QSSR} = \pi c\sqrt {(L_{k} + L_{m} )(C_{s} + C_{gap} )} $$

where *c* is the speed of light, *L*_*k*_ and *L*_*m*_ are the kinetic and magnetic inductances, respectively, and *C*_s_ and *C*_*gap*_ are the surface and gap capacitances^25^, respectively. These equivalent inductances and capacitances are functions that are dependent on the radius, width, thickness of the circular ring, in addition to the gap width and Gold plasma frequency at the resonant frequency. The $\mathop \lambda \nolimits_{QSSR}$ is substituted by 10 μm. The cross resonator is considered an electric ring resonator (ERR)^26^ that is able to strongly manipulate the incident IR radiations. The major and minor axes of the designed CEs control the electric response while the magnetic response is tuned by the thickness of the dielectric layer^27^. The resonant frequency of a regular cross resonator, i.e. crossed rectangles, can be estimated from the microwave regime using the formula^28^:

2 $$ \mathop \lambda \nolimits_{CE} = l\sqrt {\pi \varepsilon_{{TiO_{2} }} ({w \mathord{\left/ {\vphantom {w {t_{m} }}} \right. \kern-\nulldelimiterspace} {t_{m} }}){\text{ ln(}}{{t_{m} } \mathord{\left/ {\vphantom {{t_{m} } w}} \right. \kern-\nulldelimiterspace} w}{)}} $$

where *l* and *w* represent the arm length and width of the cross respectively which can be replaced for simplicity by 2*a* and 2*b*, as shown in Fig. 1b. The parameter *t*_m_ is the cross thickness as depicted in Fig. 1c. *λ*_*CE*_ is desired to be close enough to that of QSRR. Therefore, it was selected to be 9.5 μm in order to allow the wide-band operation. The relative permittivity of TiO_2_ at 9.5–10 μm is almost 1.9^29^. The dimensions resulting from the above formulas are presented in Table 1. These are taken as initial values towards realizing a wide-band metamaterial perfect absorber around 10 μm. The reflectivity of the MPA structure forms the objective function *W* = (1 − *A*), where *A* is the absorptivity, for the gradient-based optimization algorithm as described below. The MPA dimensions constitute the vector of design parameters, ***u*** = [*G, R, w, a, b, t*_*d*_*, t*_*m*_]^*T*^ for this objective function. The gradient of the objective function, i.e., absorptivity, is provided for the optimizer through adjoint sensitivities to allow for faster convergence^30^. The optimization problem is given by:

**Table 1:** The initial dimensions of the MPA extracted from LC models, and optimal dimensions of the designed MPA resulted from the adjoint-sensitivity based optimization algorithm.

Parameters	*G*	*R*	*w*	*a*	*b*	*t*_*d*_	*t*_*m*_
Initial Values [nm]	5200	2000	150	1700	325	300	150
Optimal Values [nm]	5178	1670	143	1606	319	563	279
**│(∂A.x) /(A.∂x)│**							
λ = 9.7 μm	5 × 10^–3^	3.6 × 10^–3^	2.6 × 10^–3^	2.2 × 10^–2^	1.1 × 10^–3^	4.1 × 10^–3^	7.9 × 10^–4^
λ = 10 μm	2.2 × 10^–4^	5.2 × 10^–3^	2.8 × 10^–4^	1.4 × 10^–3^	9.3 × 10^–5^	3.7 × 10^–6^	2.3 × 10^–4^

The last two rows show the sensitivities of the absorptivity at the two peaks corresponding to both resonators CE, and QSRR, respectively.

3 $$\underset{{\varvec{u}}}{\mathrm{min}}\underset{i}{\mathrm{max}}W\left({\varvec{u}},{\lambda }_{i}\right), 9.7\mathrm{\mu m}\le {\lambda }_{i}\le 10\mathrm{\mu m}, i=\left\{\mathrm{1,2},3\right\},$$

where *λ*_*i*_ represent the selected wavelength points between 9.7 μm and 10 μm to maximize the absorptivity within this range.

### Magnetic resonance in CEs and QSRR to achieve wide incident-angle and polarization-insensitive perfect absorber

The resonance wavelengths and the magnitude of the absorptivity peaks depend on the polarization of the incident IR radiation. In IR rectennas, the MPA is important to absorb the randomly polarized incoming radiation. This requires the design to be polarization insensitive to overcome the polarization-dependence of conventional antennas. Therefore, the proposed MPA was designed to retain symmetry to prevent the absorption characteristics from changing under different polarization directions. It is also preferable to account for the dependence of the absorption characteristics on the angle of incidence of the impinged electromagnetic waves.

To keep the energy harvesting functionality of the perfect absorber at a wide range of incident angles, the resonance peak is required to be held around 10 μm. Therefore, the interest to excite magnetic polaritons (MPs) from both resonators around this desirable wavelength is vital^26^. The cross resonators array that is built with a layered MIM structure usually provides two bands of absorption. One of these bands is attributed to the propagation of surface plasmon polaritons (SPPs) while the other is related to MPs and occurs at longer wavelengths^27^. The SPPs are excited at the interface between the top resonator and the dielectric layer while MPs arise from the coupling mechanism between the incident IR radiations and magnetic resonances inside the absorber^23^. The periodicity of the crossed-ellipses array is directly proportional to the SPP resonance wavelength^14^. Therefore, the periodicity *G* is designed to induce SPPs resonances at a shorter wavelength far from 10 μm, thus enabling a remarkably insensitive absorption band with respect to the angle of incidence around this resonance wavelength.

The proposed metamaterial perfect absorber shown in Fig. 1 was optimally designed to retain absorption bands resulting from MPs resonances of the quadruple split-ring resonator and crossed-ellipses close together, and to create a broadband absorption around 10 μm. The symmetry of the design and the MPs excitation allow for a wide-angle absorption with a polarization-insensitive response.

## Results and discussions

### Dimensions of the quadruple split-ring resonator and crossed-ellipses

**Figure 2:**
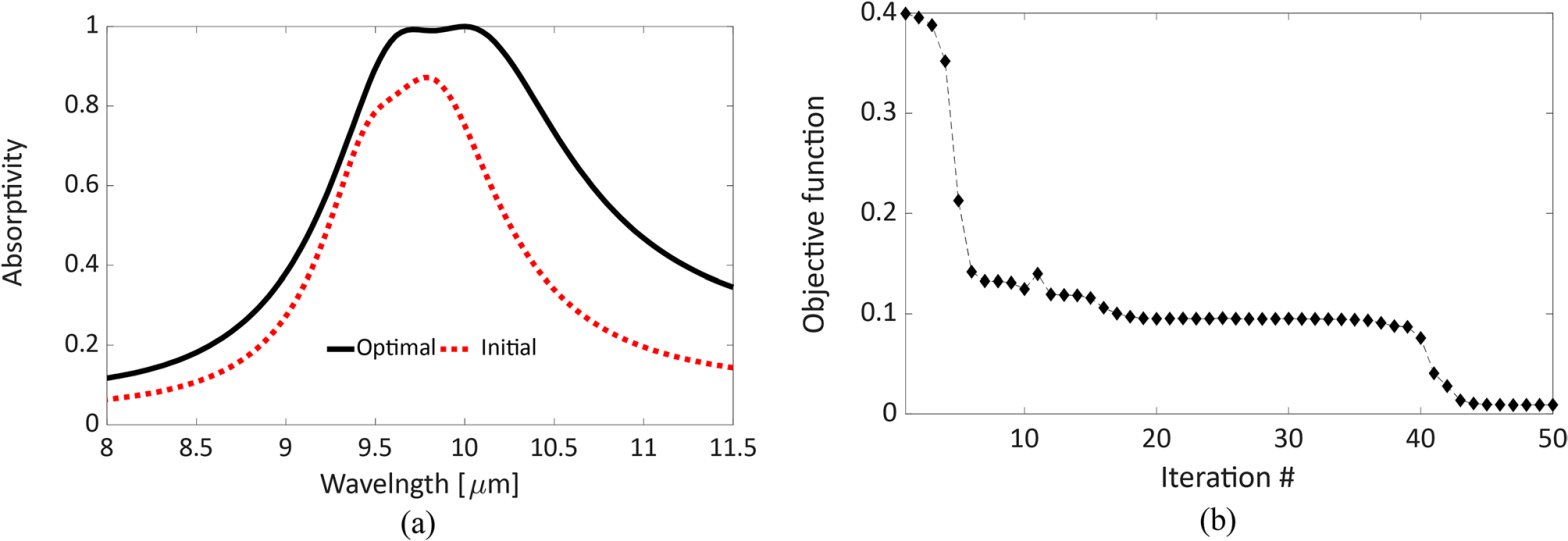
(**a**) The absorptivity of the proposed MPA structure calculated at the initial (dotted) and optimal (solid) design parameters; the bandwidth of the optimal response is ~ 1.7 times the initial one with flat near-unity absorption. (**b**) The convergence of the optimization algorithm measured with the objective function (reflectivity = 1-absorptivity) versus the iteration number. The algorithm approaches the optimal values after 43 steps.

The initial dimensions of QSRR and CE are determined using the inductor-capacitor (LC) circuit models as explained earlier. These values were used to simulate the MPA structure using the finite element method (FEM) through ANSYS HFSS. The complex permittivity of gold is defined according to the model^31^, while another model^29^ is used for TiO_2_. The absorptivity corresponding to these parameters was calculated and plotted in Fig. 2a. Although absorption peaks exist around 10 μm, their magnitude barely approaches an absorptivity of 83%.

We utilized an adjoint-sensitivity based optimization approach that is able to start from this initial design and achieve a broader band of absorption and near-unity peaks. The optimal parameters’ values are shown in Table 1 and the corresponding absorptivity is plotted in Fig. 2a. The absorption band increased from 11% initially to 18% for the optimal design. Also, the absorption band is almost flat near unity in the range between 9.6 and 10.2 μm which is equivalent to 29.4–31.3 THz. The convergence of the optimization algorithm is illustrated in Fig. 2b, where the objective function in the algorithm is defined by the absorber reflectivity. Therefore, the algorithm sought to obtain zero reflectivity, i.e. unity absorption, around the wavelength of interest. It took the algorithm 43 steps to approach this local minimum, as shown in Fig. 2b. The absorption peaks were calculated to be above 99% at 9.7 μm and 10 μm. The high absorptivity of these two wavelengths are attributed to the resonance wavelengths of each metallic resonator. The shorter resonant wavelength of 9.7 μm is attributed to the smaller resonator, i.e. crossed-ellipses, while the quadruple split-ring resonator contributes to the longer wavelength of 10 μm, as will be discussed later.

The optimization results revealed a preferred dielectric thickness, *t*_*d*_ of 0.56 μm, i.e. λ/17, which in turn allows for building thinner MPA. The ground plane thickness, *t*_*g*_, is designed to be fixed at 200 nm, which is several skin depths at this wavelength range in order to ensure approximately zero transmission. By comparing the initial and optimal values of design parameters, it is noted that the larger changes occurred mainly in 3 parameters: *R*, *a*, *t*_*d*_ which represent the inner radius of the QSRR, the ellipse’s major-axis of the CE, and the thickness of the dielectric layer, respectively. This agrees with the theoretical predictions, where in the ring resonator, the response is more dependent on the ring radius^25^. As for the cross resonator, the arm length, i.e. twice the ellipse major axis, is more dominant^28^. Also, the insulator thickness tunes the coupling within the MIM structure. Owing to all parameters’ variations, the absorption band was tailored efficiently, and hence the required performance was realizable.

By taking fabrication tolerances into account at this nanometer scale, the absorption sensitivities with respect to all design parameters were calculated at the two wavelengths of 9.7 μm and 10 μm. These sensitivities, obtained through adjoint sensitivity calculations by considering 10% change in each parameter, are presented in Table 1. Most of the values are relatively small which in turn enables acceptable performance at predictable and unavoidable tolerances. From the last two rows in Table 1, the largest expected variation in the absorptivity at the 9.7 μm peak, which is 0.022, is attributed to the ellipses’ major axis, *a*, while the other peak at 10 μm is almost insensitive. Moreover, a slight change of 0.004, at this shorter wavelength peak, is associated with the 10% change in the thickness of the dielectric layer with almost no impact at the longer wavelength. As for the longer wavelength peak, a relatively small change of 0.005 is expected due to variations in the radius of the ring resonator, with lesser impact on the shorter wavelength peak.

### Electromagnetic field distributions at MP resonances of the polarization insensitive MPA

**Figure 3:**
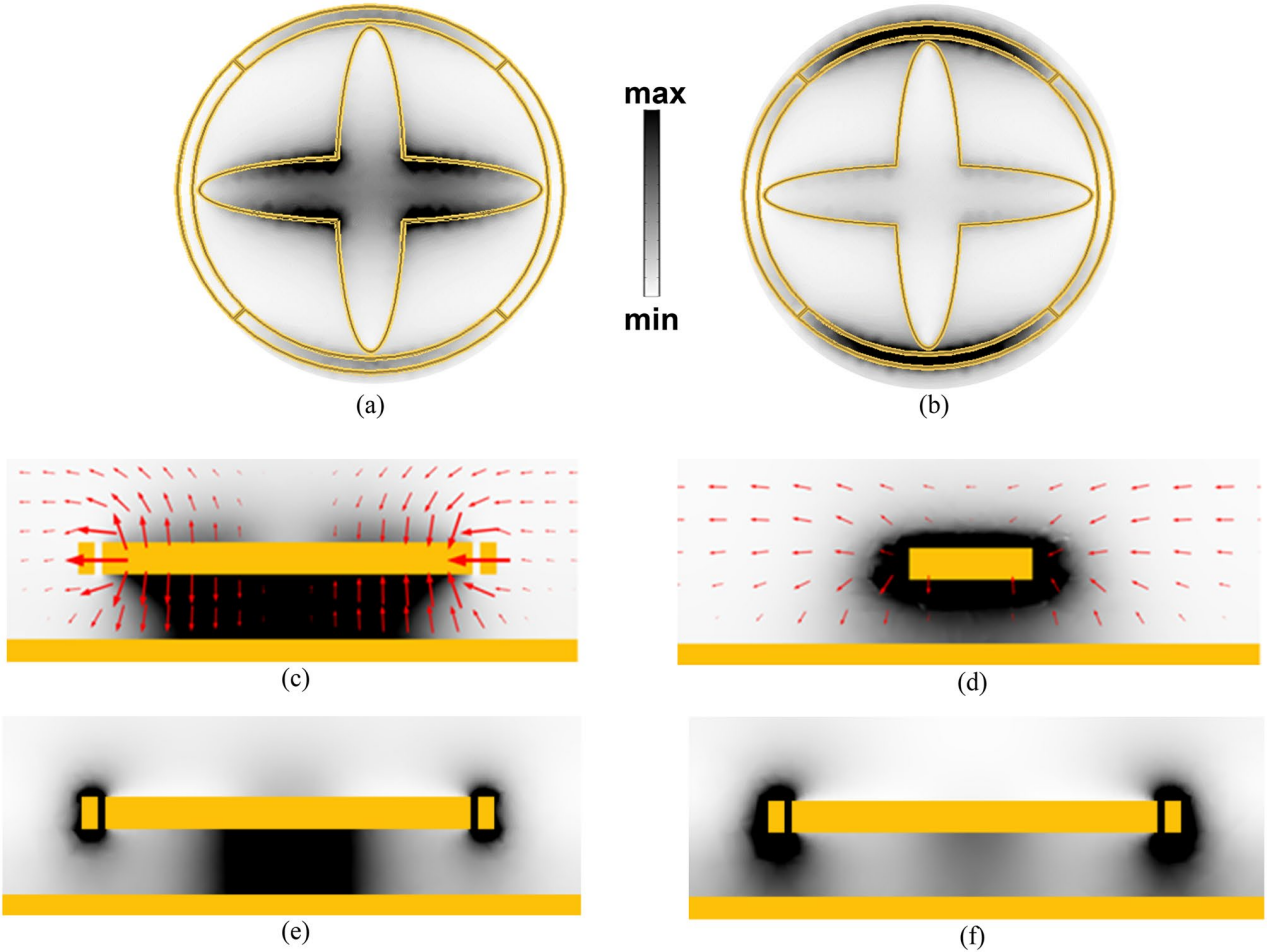
The distribution of the magnetic field intensity |H|^2^ of the proposed MPA structure calculated at two peaks of 9.7 μm and 10 μm, respectively. (**a**, **b**) at the cross section at the interface between the top resonators and the dielectric layer below, where the darker color represents higher magnetic field intensity, all plots at the same scale. (**c**, **d**) the grey scale spectrum maps the magnetic field intensity at XZ-plane cross-section, while the red arrows show the electric field distribution at both resonance wavelengths emphasizing the creation of magnetic resonances. The cross-section is cut along the CE arm in (**c**), while it passes through the circumference of the QSRR in (**d**). (**e**, **f**) YZ-plane cross-section illustrates the dominant source of the resonance occurs at each absorption peak; the left one shows the field beneath the crossed ellipses resonance is dominating, while the right one reveals the domination of QSRR resonance. (ANSYS HFSS 2018, https://www.ansys.com/, COMSOL Multiphysics 5.3 ).

We calculated the magnetic field distributions in the cross-sections of the MPA structure at the wavelengths of 9.7 μm and 10 μm, where the absorption peaks reach 99.99%. These calculations were obtained using FEM simulations in both ANSYS HFSS and COMSOL Multiphysics, and the corresponding field distributions are presented in Fig. 3(a-f). In the simulations, a normal incident electromagnetic wave impinged the MPA structure directed from top to bottom, i.e. *z*-direction, with the electric field polarized in the *x*-direction. The magnetic field intensity |H|^2^ was investigated in the XY plane, parallel to the top cross-section at the interface between the top gold resonators and the TiO_2_ dielectric layer underneath. Figures 3a,b show the magnetic resonances at 9.7 μm and 10 μm, respectively. The magnetic field is confined around the arm of the crossed ellipses that is parallel to the incident electric field at 9.7 μm, as depicted in Fig. 3a. At 10 μm, the magnetic field is concentrated within the top and bottom parts of the QSRR, as shown in Fig. 3b.

Figures 3c,d illustrate the electric field and magnetic field distributions across the XZ plane cross-sections. At 9.7 μm, the electric field vectors form circles that exhibit a strong magnetic resonance that is shown in Fig. 3c. At the other wavelength of 10 μm, the XZ cross-section was selected at the perimeter of the QSRR, where the electric fields are arranged to compose a confined magnetic resonance around the QSRR, as illustrated in Fig. 3d. The YZ cross-sections plotted in Figs. 3e,f show an enhanced magnetic field in the dielectric layer below each top resonator according to Lenz’s law. The magnetic field confinement shown in Fig. 3a–e indicates the strong MPs excited by the CE and the QSRR at 9.7 μm and 10 μm, respectively.

### Electric field enhancement in the gaps of the metamaterial perfect absorber and energy harvesting capabilities

**Figure 4:**
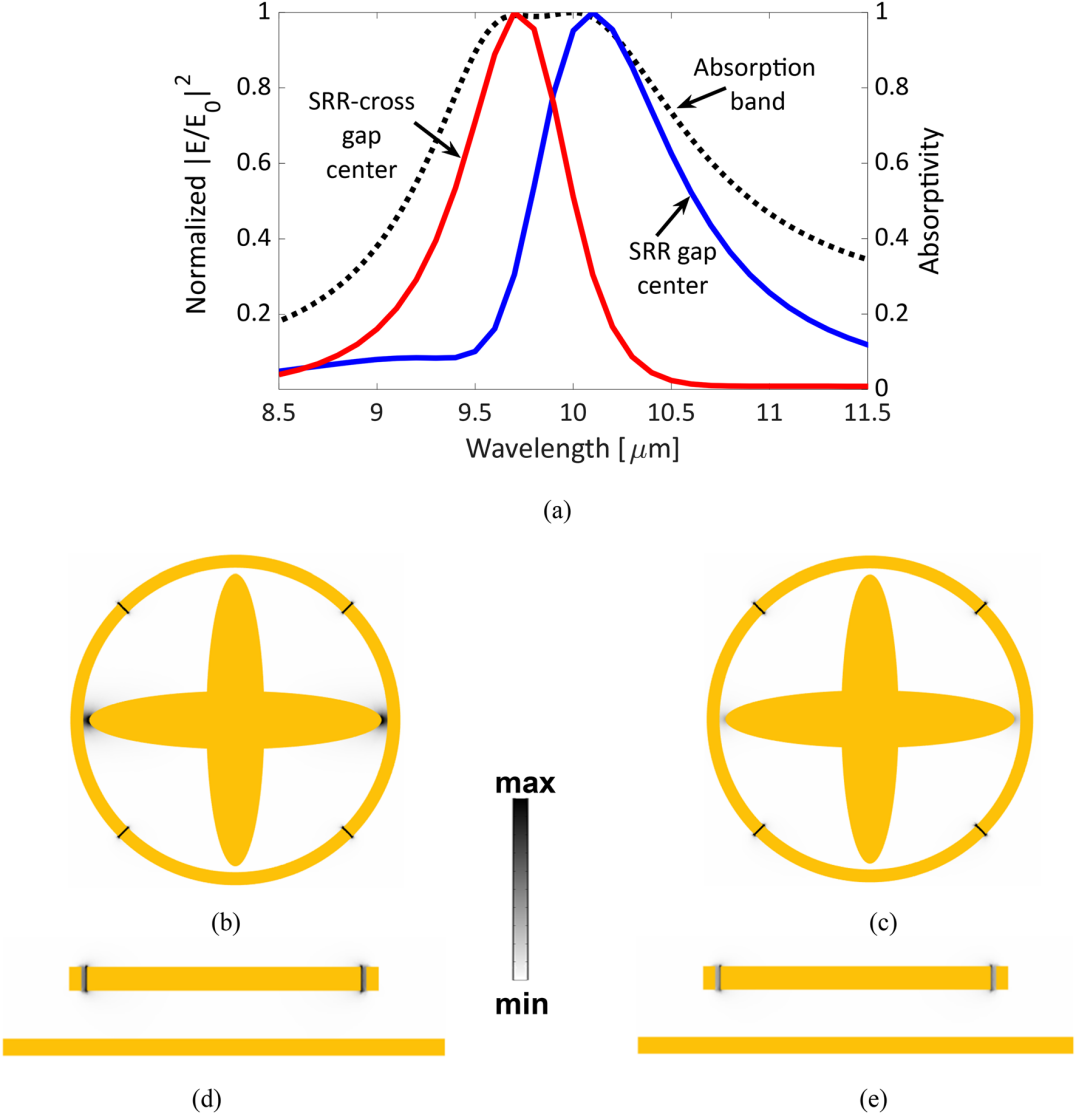
(**a**) The absorptivity of the optimal MPA design in dotted black line and the normalized electric field intensity enhancement |E/E_0_|^2^ at the center of the gap between the CE and QSRR and center of the slits of the QSRR in red and blue solid lines, respectively. The electric field intensity distribution at 9.7 μm and 10 μm, respectively, (**b**, **c**) through the XY cross-section, and (**d**, **e**) through the YZ cross-section. (COMSOL Multiphysics 5.3 https://www.comsol.com/).

The designed metamaterial perfect absorber is featured with multiple gaps that promote the integration with diodes, i.e. rectifiers. This integration allows the MPA to harvest the absorbed energy from IR radiation and simultaneously transform it into a useful DC current. The electric field intensity enhancement |E/E_0_|^2^ at the center of each gap is depicted in Fig. 4a. The gaps are categorized into two groups; the gaps between the crossed ellipses arms and the surrounding ring resonator, and the other gaps formed by splits of the ring resonator itself. The electric field enhancement of the first category is blue shifted, as expected from the resonance characteristics resulting from the crossed ellipses which are around 9.7 μm as shown in Fig. 4a. The electric field enhancement at the center of the other gaps inside the ring resonator’s splits is confined almost around 10 μm as presented in the same figure. These electric field intensities are normalized and plotted with the absorptivity of the proposed metamaterial perfect absorber. The figures illustrate the capability of each group of those tiny gaps to confine the absorbed fields. Both groups of gaps work simultaneously to trap the incoming IR radiations and cover the whole absorption band as shown in Fig. 4a.

The distribution of the electric field intensity is plotted in Fig. 4b–e. The top views of the electric field intensity in the XY plane of PMA structure exhibit a strong electric field confinement at the gaps located between the CE arms and the surrounding QSRR combined with a weaker resonance inside the splits of QSRR at 9.7 μm, as depicted in Fig. 4b. While the electric field is mostly confined inside the splits of QSRR at 10 μm as presented in Fig. 4c. Figure 4d–e illustrate the electric field confinement distribution across the YZ plane cross section which agrees with the previous results, where the electric field is noticeably confined inside the gaps between CE arms and QSRR inner perimeter at the shorter wavelength of 9.7 μm.

### Energy harvesting using MPA combined with MIM diodes

**Figure 5:**
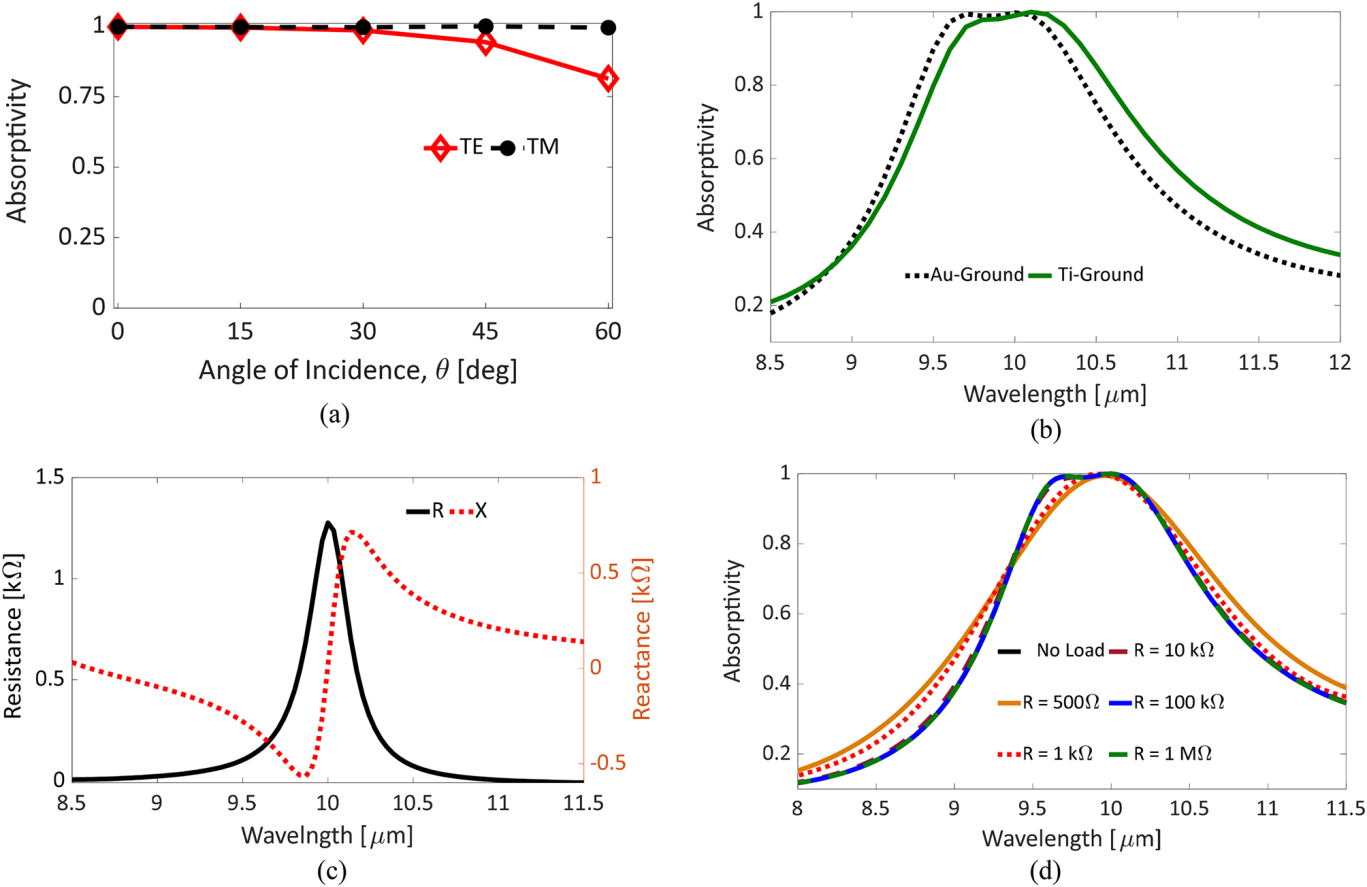
(**a**) Incident angle sensitivity of the absorptivity of the optimal MPA structure through calculating absorptivity peaks for TE and TM polarizations. (**b**) The absorptivity of the optimal MPA structure using a gold ground compared to the titanium. (**c**) The resistance and reactance of the MPA structure, (**d**) Diode’s resistance sensitivity, resistances range from 500 Ω up to 1 MΩ.

The strong electric field confined inside the MPA gaps promotes its applicability and suitability to efficiently replace the nano-antennas in the rectenna system for energy harvesting. The developed MPA design offers a polarization insensitive performance due to the symmetry. Owing to the excitation of MPs through both top resonators, i.e. CE and QSRR, the proposed MPA exhibits almost insensitive absorption peaks to incident angle variations up to 60°, as illustrated in Fig. 5a. The shift in the resonance wavelength in this range is at most 2%, which falls in the absorption band of interest. These characteristics support the use of the proposed MPA rather than regular nano-antennas that lack at least one of these features, for thermal energy harvesting.

The metal–insulator-metal (MIM) diodes are the best candidate that can work with the proposed perfect absorber in the IR region. Due to the dominance of the tunneling current through thin dielectric barriers with a few nanometers thickness, the MIM diodes can rectify the ultra-high frequency AC signal captured by the MPA structure. Additionally, the fabrication of the MPA structure combined with an MIM diode would reduce the complexity as compared to the integration of MIM diodes with nano-antennas. To reduce the complexity of the manufacturing, we designed the metallic ground plane to be made of titanium instead of gold. The resulted absorptivity of the Ti-based MPA structure is compared to the Au-based one as depicted in Fig. 5b. A slight shift in absorption band due the change of the ground materials is noticed because of different optical permittivities. In addition, a small increase in the absorption bandwidth is achieved. The choice of Ti as a ground plane facilitates forming the TiO_2_ layer on top of it.

One of the main shortcomings of recent IR rectennas is the impedance mismatching between the MIM diode and the nano-antenna. The MIM diodes possess a wide range of diode resistance from few hundreds up to several Mega Ohms^8^ depending on the materials’ properties of the diode including barrier heights, dielectric constants and their corresponding thicknesses, and area^9^. Nano-antennas, on the other hand, generally have a small resistance in the range of few tens of ohms^9^. The diode’s resistance is considered the load of the nano-antenna and thus a small portion of the power received by the nano-antenna can reach the diode terminals to be rectified. Experimental attempts were carried out to improve this matching by selecting diodes’ dielectric materials with ultra-thin thicknesses to achieve smaller diode’s resistance. However, this matching should be accomplished through both rectenna elements, i.e. diode and nano-antenna, simultaneously. We calculated the impedance of our MPA structure and presented the results in Fig. 5c. The resistance is greater than 500Ω over the absorption bandwidth, which is higher than that of the dipole and bowtie nano-antennas^32^. This consequently improves the matching efficiency^9^.

**Figure 6:**
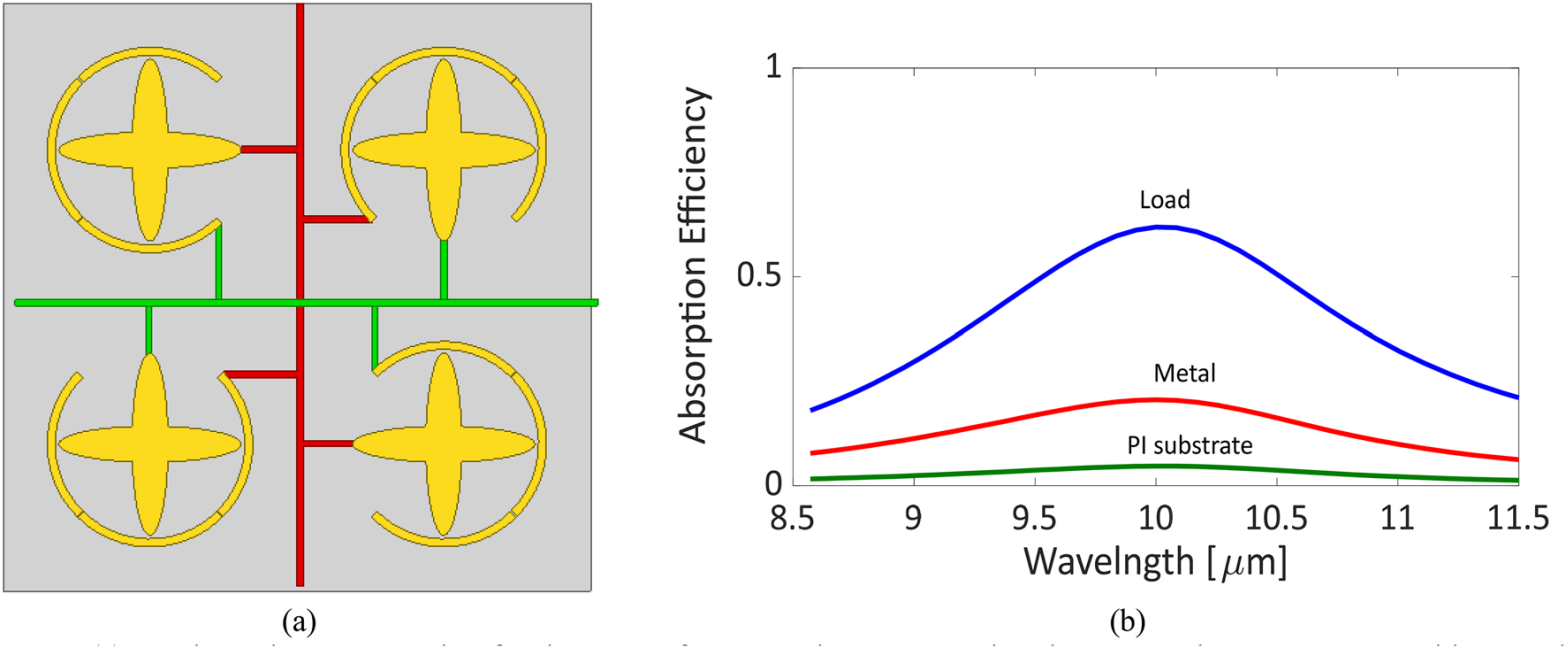
(**a**) A schematic representation for the array of connected rectennas using the proposed MPA structure with control lines in green (horizontal) and red (vertical), (ANSYS HFSS 2018, https://www.ansys.com/) (**b**) The absorption efficiency of the proposed MPA structure calculated for the substrate (green), load (blue) and metal (red) of the MPA with PI substrate.

The wide range of possible diode’s resistance drives the investigation of the sensitivity of the proposed MPA absorptivity to this range of resistances. The MIM diode’s resistance was varied from a 500 Ω up to 1 MΩ. The absorptivity shows insensitive response for diode resistances greater than 1 kΩ as illustrated in Fig. 5d. For resistances of 1kΩ or less, the rectenna shows a perfect absorption but with a little reduction in the flat absorption band while the absorption bandwidth increased. Additionally, the MIM diodes are connected in series through the proposed rectenna-like design and consequently decrease the capacitance of the equivalent diode. This reduction in turns allows for attaining a shorter time constant and hence a higher cut-off frequency. This also leads to further improvement in the rectenna performance. The final rectenna structure will employ the proposed MPA with the MIM diode as a unit cell in a large 2D array with the corresponding interconnections as represented in Fig. 6a.

The absorbed power per each layer of the MIM structure can be determined by considering the corresponding complex permittivity of each material at this wavelength range^33^. Therefore, the absorption efficiency can be calculated as the ratio of power absorbed by each layer to the total absorbed power^34,35^. By considering that the extinction coefficient, *k*, of TiO_2_ is ~ 0.14 around 10 μm wavelength^29^, the power loss in the substrate is expected to be large and will dominate the absorption efficiency. However, building the MIM structure using a low-loss substrate such as polyimide (PI), where *k* ~ 20 × 10^–3^, allows for a drastic reduction in substrate losses compared to TiO_2_ case^36^. The absorption efficiency is estimated using the PI layer and presented in Fig. 6b, where most of the absorbed power is delivered to the load.

The proposed metamaterial perfect absorber (MPA) design in this work is built as a metal–insulator-metal (MIM) structure composed of a crossed ellipse (CE) concentric with a quadruple split-ring resonator (QSRR). The structure is optimized, around 10 μm, using an adjoint sensitivity approach to achieve near unity wide-band absorptivity. The proposed MPA supports wide reception-angle up to 60°. The strong electric field enhancement inside the gaps of the structure allows for simple integration with MIM diodes to form a complete rectenna for thermal energy harvesting around 10 μm. The optimal design shows insensitive performance with different MIM diode’s resistances, and accordingly provides more flexibility in selecting the diodes’ materials. Finally, this proposed work offers a scalable approach to design MPA at different resonance wavelengths.

## Methods

### Numerical simulations

We employed an LC circuit model to determine the initial parameter values for the wide incident-angle and polarization insensitive MIM absorber structure. An FEM method using ANSYS HFSS simulator software was used to verify the circuit model results. It was integrated within a MATLAB-based optimization algorithm. At each optimization step, the absorptivity and the corresponding adjoint sensitivities with respect to all design parameters were calculated using ANSYS HFSS and then passed to MATLAB to move forward. Another FEM model was built in COMSOL Multiphysics to study the performance at different incident angles. Periodic boundary conditions were applied in *x* and *y* directions of the unit cell with perfectly matched layer in the top of the structure along z-axis. The incident IR consisted of TM-mode plane waves. The mesh was set to be with a minimum 2 nm in the vicinity of the tiny gaps and inside the top metal structures.
